# Feed-forward neural network model for hunger and satiety related VAS score prediction

**DOI:** 10.1186/s12976-016-0043-4

**Published:** 2016-07-07

**Authors:** Shaji Krishnan, Henk F. J. Hendriks, Merete L. Hartvigsen, Albert A. de Graaf

**Affiliations:** Risk Analysis for Products In Development, TNO, Utrechtseweg 48, P.O. Box 360, Zeist, 3700 AJ The Netherlands; Department of Endocrinology and Internal Medicine, Aarhus University, Tage-Hansens Gade 2, Aarhus C, DK-8000 Denmark; Top Institute Food and Nutrition, Nieuwe Kanaal 9A, Wageningen, 6709 PA The Netherlands

**Keywords:** Satiety, Visual analog scales (VAS), Modeling

## Abstract

**Background:**

An artificial neural network approach was chosen to model the outcome of the complex signaling pathways in the gastro-intestinal tract and other peripheral organs that eventually produce the satiety feeling in the brain upon feeding.

**Methods:**

A multilayer feed-forward neural network was trained with sets of experimental data relating concentration-time courses of plasma satiety hormones to Visual Analog Scales (VAS) scores. The network successfully predicted VAS responses from sets of satiety hormone data obtained in experiments using different food compositions.

**Results:**

The correlation coefficients for the predicted VAS responses for test sets having i) a full set of three satiety hormones, ii) a set of only two satiety hormones, and iii) a set of only one satiety hormone were 0.96, 0.96, and 0.89, respectively. The predicted VAS responses discriminated the satiety effects of high satiating food types from less satiating food types both in orally fed and ileal infused forms.

**Conclusions:**

From this application of artificial neural networks, one may conclude that neural network models are very suitable to describe situations where behavior is complex and incompletely understood. However, training data sets that fit the experimental conditions need to be available.

## Background

Decades of studies indicate that hunger and feeding cycles involve complex hormonal and neuronal signal interactions, including e.g., hormone releases from the gastro-intestinal tract and neuro-endocrine signals from peripheral organs like liver, pancreas, and stomach to the brain [1]. Satiety is that satisfactory feeling of fullness attained as a result of feeding [2]. The hormones released by the gastro-intestinal tract associated to satiety, have also been subject of numerous obesity-and diabetes-related studies [3, 4]. Although there are many hormones relating to hunger and satiety [5], the three key hormones appear to be the glucagon-like peptide-1 (GLP-1), cholecystokinin (CCK), and peptide YY (PYY) [6, 7]. GLP-1 is mainly secreted by the distal part of the small intestine, the ileum [8], whereas CCK is secreted in the duodenum and PYY in the ileum [9, 10].

The amount of satiety hormones released from the gastro-intestinal tract, and thus their satiating effects, are related to the composition and the amount of nutrient ingested [11, 12]. High protein diets produce higher satiating effects than low protein diets [13]. Some evidence suggests that dietary protein is more satiating than carbohydrate or fat [14, 15]. Also, there are location specific satiety effects of nutrients along the gastro-intestinal tract. Particularly, activating the distal part of the gut, the ileum, produced the maximal satiety effects in terms of reduction in hunger and food intake [16]. This neuro-endocrine negative feed back signaling scheme that results in producing satiating affects is known as the ileal brake [17, 18].

One of the most commonly used indices for sensations for appetite is the Visual Analog Scales (VAS) [19]. The VAS scores evaluate desire to eat, hunger, fullness and satiety. These VAS scores on a 100 pt scale are marked on a 100 mm long paper with 0 and 100 corresponding to a low and a high appetite respectively. For example, 0 (low) score for hunger meant that the subject is not at all hungry, while a 100 (high) meant that the subject is extremely hungry [20]. The reproducibility, power and validity of this index have been well established [21, 22].

Modeling a complex physiological system that predicts the VAS response curves relating to appetite sensation in a deterministic or a mechanistic form is a complicated task. However, machine learning methods like an artificial neural network (ANN) can be applied to learn the patterns of VAS response curves given satiety signal measurements such as time courses of satiety hormone concentrations. Neural network modeling is one of the most promising modeling techniques with many applications in biology and medicine [23–28].

ANNs are simple abstractions of biological neurons realized on a computer as a software program. Similar to a biological neural network, ANN consists of computer programmed processing units (nodes) that are interconnected in a way that signals from the input travel through the interconnected nodes to the output. Once an ANN is realized on a computer, it can be trained with appropriate data to create useful input-output transfer functions, and thus various applications [29].

Some of the compelling reasons to use ANNs for modeling complex behavior as VAS response curves relating to appetite sensation are that they do not require mechanistic details of the underlying physiology, are workable with available data, and allow for immediate predictions. To our knowledge, ANN’s have not yet been applied to predict hunger and satiety related VAS scores in humans with an exception to a statistical model that quantitatively estimate the duration of human satiety response time [30]. Although this statistical model is capable to estimate the duration of VAS response score for hunger to return to baseline pre-prandial levels after a meal consumption, it may not be able to reflect the observed hunger response profile due to the restrictions imposed by the parameters of the underlying distribution. In contrast, the ANN’s have the freedom to choose parameter values that closely approximate the VAS response curves at every observed time point.

The objectives of the present paper are to i) build a neural network model that learns VAS response patterns for desire to eat, hunger, fullness, and satiety from GLP-1, CCK and PYY satiety hormone measurements, and ii) use this trained neural network model to predict VAS response patterns for desire to eat, hunger, fullness, and satiety for a given set of GLP-1, CCK and PYY satiety hormone measurements. The results of such a prediction will be considered acceptable if i) the correlation coefficients of the prediction and the measured VAS responses are above 0.85, and ii) the predicted VAS responses can discriminate the satiety effects of varying food composition types fed either orally or gastrointestinally infused.

## Methods

### Multilayer feed-forward neural network

For the purpose of predicting hunger and appetite related VAS responses, a multilayer feed-forward neural network was built using the MATLAB^®;^, Neural Toolbox^™^, R2013b [31]. The toolbox also implements cross validation procedures, and hence is robust in construction and not prone towards over-fitting. One of the reasons to choose a multilayer feed-forward neural network is for its comprehensive foundation and is one of the widely used models in many practical applications [32]. In addition to this, the performance of a multilayer neural network degrades gracefully in the presence of increasing amounts of noise. One known disadvantage however is its lengthier training time [33].

**Fig. 1:**
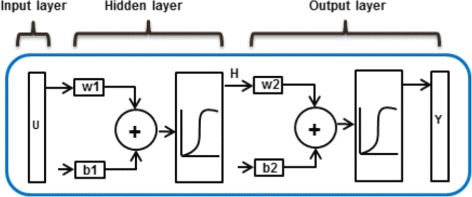
A multilayer feed-forward neural network. A multilayer feed-forward neural network consisting of an input layer, an output layer, and a hidden layer. A weighted and biased input is non-linearly transferred with a log-sigmoid function by the hidden layer as an input for the next layer which again is weighted, and biased and non-linearly transferred to the output. Input U, weights w1 and w2, and output Y are multicomponent vectors, while biases b1 and b2 are scalar

A multilayer feed-forward network consists of an input layer of nodes an output layer of nodes, and one or more hidden layers (see Fig. 1). The hidden layers are placed between the input and the output layer. Each layer consists of one or more processing nodes. The output of the node from one layer are connected to one or more nodes of the next layer. Each node implements a weighted (**w**) sum of its inputs (**U**) and a bias (b) which is then non-linearly transfered to one or more nodes of the next layer. Here, U and w are vectors i.e contain multiple components. Thus for the given example in Fig. 1, a weighted and biased input (**U**∗**w1**+b1) is non-linearly transfered with a log-sigmoid function (**H**=logsig(**U**∗**w1**+b1)), also known as the activation function, by the hidden layer as an input for the next layer which again is weighted, and biased and non-linearly transfered to the output (**Y**=logsig(**H**∗**w2**+b2)). One of the reasons to choose a log-sigmoid function is because the return values of this function is bounded and thus the magnitude of the parameters do not grow extremely large.

Before using the network for predictive or classification purposes, the multilayer feed-forward network (or any ANN) needs to be trained. The training is achieved by feeding the network with sets of input-output data called the training set. The objective of training is to estimate the weights and bias values at every node of the network such that the trained network satisfactorily relates every input-output data from the training set. Such a trained multilayer feed-forward neural network is capable to compute a unique output for wide range of inputs.

The input-output training set of the multilayer feed-forward neural network contained satiety hormone: GLP-1, CCK, PYY time resolved measurements as inputs and the time resolved VAS scores for hunger, fullness, desire to eat, and satiety as outputs, taken from a number of experimental studies. To capture the interaction among the three independent time resolved satiety hormone measurements in determining the four VAS responses, both in temporal (t) and spectral (*ω*) domain, the inputs were modified before training the network. In the spectral domain, the power spectral density estimates, as a function of frequency (*ω*), were calculated via the Thomson multitaper method as described in Du et al. [34]. Thus effectively, the inputs (**U**) and the outputs (**Y**), both vectors, used to train the multilayer feed-forward neural network model in predicting the hunger and appetite related VAS response from satiety hormones were as shown in Eq. 1.

1 $$\begin{array}{@{}rcl@{}} \mathbf{U} & = & [I(t), M(t), I(\omega), M(\omega)] \\ \mathbf{Y} & = & [V_{d}(t), V_{h}(t), V_{f}(t), V_{s}(t)]  \end{array} $$

where I(t), M(t) are the satiety hormone measurements and their mixed effects (see Eq. 2) in the temporal domain and I(*ω*), M(*ω*) are the power spectral density estimates of the satiety hormone measurements and their mixed effects (see Eq. 3) in the spectral domain. Similarly, *V*_*d*_(*t*),*V*_*h*_(*t*),*V*_*f*_(*t*),*V*_*s*_(*t*), are the VAS response for desire to eat, hunger, fullness, and satiety measurements in the temporal domain.

2 $$\begin{array}{@{}rcl@{}} I(t) & = & [G(t), C(t), P(t)] \\ M(t) & = & [G(t)*C(t), G(t)*P(t), C(t)*P(t), G(t)*C(t)*P(t)]  \end{array} $$

3 $$\begin{array}{@{}rcl@{}} I(\omega) & = & [G(\omega), C(\omega), P(\omega)] \\ M(\omega) & = & [G(\omega)*C(\omega), G(\omega)*P(\omega), C(\omega)*P(\omega), G(\omega)*C(\omega)*P(\omega)]  \end{array} $$

where G, C, and P are the satiety hormone GLP1, CCK, and PYY measurements respectively.

A multilayer feed-forward neural network model, with an input, an output, and 2 hidden layers trained, with sufficient amount of input-output datasets will instantly predict the VAS response for desire to eat, hunger, fullness, and satiety for an arbitrary set of GLP-1, CKK, and PYY values measured in time. However, in practice two problems can arise, i) not for all of the three satiety hormones, data are available ii) hormones are not measured in the same sampling scheme as in the training set. Since these two problems essentially amount to a missing data problem they are addressed as follows,

In the case of missing hormone data an optional multilayer feed-forward neural network with an input, an output, and 2 hidden layers was implemented and appropriately trained to predict the missing hormones set from the data set of measured satiety hormones. The input and output of such a neural network is shown in Eq. 4. When provided with one or more sets of satiety hormones, where each set contained all satiety hormones, GLP1, CCK, and PYY from the same experimental study, the inputs and the outputs of the neural network were iteratively trained with one satiety hormone from each set at the input and all the three hormones from the same set at the output. Hence the number of training sessions for each set were three. Now given an arbitrary satiety hormone, either GLP1, or CCK, or PYY, the trained network predicted the rest (in practice all the three satiety hormones).

4 $$\begin{array}{@{}rcl@{}} \mathbf{U} & = & [X(t)] \\ \mathbf{Y} & = & [G(t), C(t), P(t)]  \end{array} $$

where X(t) is any of the satiety hormone GLP1, or CCK, or PYY measurements in the temporal domain, and G(t), C(t), and P(t) are the satiety hormone GLP1, CCK, and PYY measurements in the temporal domain respectively.

Similarly, in the case of filling in additional satiety hormone data outside the sampling interval, a dynamic neural network implementing an Nonlinear Autoregressive Neural Network (NARX) [31], capable to predict the next time series data from its previous time series inputs and outputs, was included. The input and output of such a neural network is shown in Eq. 5. This neural network when provided with a shorter time sequence of a satiety hormone data, predicted the rest of the sequence until a pre-specified length.

5 $$\begin{array}{@{}rcl@{}} \mathbf{U} & = & [X(t-L)] \\ \mathbf{Y} & = & [X(t)]  \end{array} $$

where X(t) is any of the satiety hormone GLP1, or CCK, or PYY measurements in the temporal domain, and L is the time lag.

### Description of datasets: Ileal infusion

Datasets for modeling included data from thirteen volunteers that were included in an experimental study performed by the Top Institute Food and Nutrition (TIFN) to assess the effect of different macro-nutrients on ileal brake activation^1^ [35]. Briefly, thirty minutes after breakfast, a solution containing either saline (placebo) or safflower oil ((SO), 51.7 kcal) or Sucrose-low (17.2 kcal) or Sucrose-high (51.7 kcal) or Casein-low (17.2 kcal) or Casein-high (51.7 kcal) was infused into the ileum using a catheter. From the volunteers, blood samples and VAS for desire to eat, hunger, fullness and satiety, were collected at various time points after breakfast. From each blood sample, the plasma concentrations of satiety hormones GLP1, CCK, and PYY were measured.

The training set and the test set chosen from this study for neural net training and testing purposes are shown in Table 1. The training set, Dataset A, had 2 subsets of data, while the test set, Dataset B, had three subsets of data. Each subset contained satiety hormone and VAS measurements from 13 volunteers. In the training set, measurements for all satiety hormones required for the neural net training were available, while in the test set, data for the satiety hormone, CCK were missing.

**Table 1:** Description of datasets: Ileal infusion, training set: Dataset A, and the test set: Dataset B, employing ileal macronutrient perfusion [35]

Data Source	Data Name	G	C	P	H	S	F	D	Sub	*T*_*s*_	*T*_*e*_	Ml/If	NPS
Dataset A	Placebo	✓	✓	✓	✓	✓	✓	✓	13	–15	240	0, 30–120	10
	Safflower	✓	✓	✓	✓	✓	✓	✓	13	–15	240	0, 30–121	10
Dataset B	Casein-high	✓	✓	✓	✓	✓	✓	✓	13	–15	240	0, 30–122	10
	Casein-low	✓	✗	✓	✓	✓	✓	✓	13	–15	240	0, 30–123	10
	Sucrose-high	✓	✗	✓	✓	✓	✓	✓	13	–15	240	0, 30–123	10

The letters or abbreviation in the headers are as follows: DataName = the perfusion macro-nutrient type; G = GLP-1 (pmol/l); C = CCK (pmol/l); P = PYY (pg/ml); H = VAS for Hunger; S = VAS for Satiety; F = VAS for Fullness; D = VAS for Desire to eat; Sub = Number of subjects; Ts = Tstart (min); Te = Tend (min); Ml/If = time point/range for Meal/Infusion; NPS = Number of Plasma Samples. A check mark (✓) indicates that the corresponding satiety hormone measurements and/or VAS scores were available, while a cross mark (✗) indicates non-availability of the measurements

### Description of datasets: Oral intake and gastric infusion

As a second training set, Data set C, (Table 2) generated from a study performed by the TIFN at the Wageningen University & Research centre [36] was chosen. The intention of the study was to measure the effect of gastric processing of liquid fat formulations on the timing of the release of the fat to the small intestine and the effect of this on the time evolution of intestinal hormone release into blood circulation and how this relates to the evolution of feelings of hunger and satiety.

**Table 2:** Description of datasets: Oral intake and gastric infusion, Dataset C, and the test set: Dataset D. Dataset C used meals and gastric infusions, whereas dataset D only used meals

Data Source	Data Name	G	C	P	H	S	F	D	Sub	*T*_*s*_	*T*_*e*_	Ml/If	NPS
Dataset C	ho	✓	✓	✓	✓	✓	✓	✓	15	–10	180	30	9
	hs	✓	✓	✓	✓	✓	✓	✓	15	–10	180	30	9
	uhs	✓	✓	✓	✓	✓	✓	✓	15	–10	180	30	9
Dataset D	d3	✓	✗	✗	✓	✓	✓	✓	15	0	270	0	10
	d4	✓	✗	✗	✓	✓	✓	✓	15	0	270	0	10

The letters or abbreviation in the headers are as follows: DataName = macro-nutrient type or intake form; G = GLP-1 (pmol/l); C = CCK (pmol/l); P = PYY (pg/ml); H = VAS for Hunger; S = VAS for Satiety; F = VAS for Fullness; D = VAS for Desire to eat; Sub = Number of subjects; Ts = Tstart (min); Te = Tend (min); Ml/If = time point/range for Meal/Infusion; NPS = Number of Plasma Samples. A check mark (✓) indicates that the corresponding satiety hormone measurements and/or VAS scores were available, while a cross mark (✗) indicates non-availability of the measurements

To this end, 15 volunteers received 500 ml dosages of 8 % sunflower oil, 0.4 % Tween 80 and 91.6 % water, corresponding to an energy content of 360 kcal. These dosages were given orally (homogenised oral(ho)), by gastric infusion (homogenised stomach(hs)) or in the form of a gastric infusion of water followed by the oil phase (unhomogenised stomach(uhs)). Blood samples were collected at various time points to measure GLP-1, CCK, and PYY concentrations [36].

As a second test set, Data set D, (Table 2) generated in a study performed at Aarhus University Hospital, Aarhus, Denmark, on 15 Caucasian subjects having the metabolic syndrome to investigate the impact of arabinoxylan, *β*-glucan and whole grain rye compared with refined wheat on glycaemia and satiety^2^ [37] were chosen. The study employed meals of refined wheat breads supplemented with concentrates of arabinoxylan (data1(d1)), or concentrates of *β*-glucan (data2(d2)), whole grain rye bread (data3(d3)) and refined wheat bread (data4(d4)). After the meal intake, blood samples were drawn from volunteers at various time points in addition to assessment of appetite sensation (VAS for desire to eat, hunger, fullness, and satiety). From each blood sample, the plasma concentration of the satiety hormone GLP-1 was measured.

## Results and discussion

In the following sections the results of applying the respective trained neural networks in VAS response prediction for the various test sets shown in Tables 1 and 2 will be discussed.

### Predicting VAS profiles for test sets with ileal infusion (complete satiety hormone data)

**Fig. 2:**
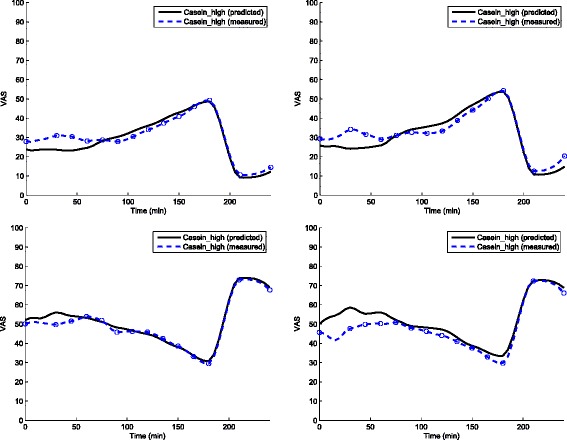
Measured and predicted VAS profile. Measured and predicted VAS profile (clockwise from top-left): Desire to eat, Hunger, Fullness, Satiety and for test set Dataset B: Casein-high infusion averaged over 13 volunteers. The respective correlation coefficients for Desire to eat, Hunger, Fullness, Satiety are 0.96, 0.95, 0.95, and 0.98

All satiety hormone measurements were available for Dataset B with the DataName: Casein-high. The VAS profiles for hunger, fullness, desire to eat, and satiety predicted from measured GLP-1, PYY, and CCK are shown in Fig. 2. The respective correlation coefficients for Desire to eat, Hunger, Fullness, Satiety were 0.96, 0.95, 0.95, and 0.98.

### Predicting VAS profiles for test sets with ileal infusion (incomplete satiety hormone data)

**Fig. 3:**
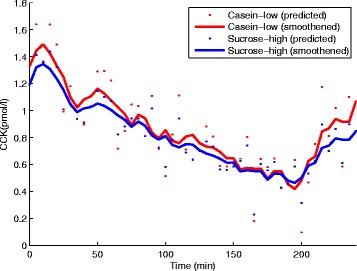
Predicted CCK response. Predicted CCK response averaged over 13 volunteers for Dataset B: Casein-low and Sucrose-high infusions. The dots indicate the predicted CCK concentrations for individual volunteers at various time points. The dots are connected and smoothened to represent the respective CCK concentration over time

**Fig. 4:**
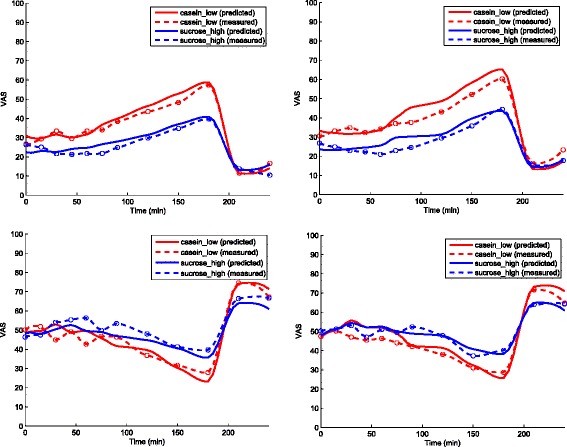
Measured and predicted VAS profile. Measured and predicted VAS profile (clockwise from top-left): Desire to eat, Hunger, Fullness, Satiety for test set Dataset B: Casein-low and Sucrose-high infusions averaged over 13 volunteers. The respective correlation coefficients for Desire to eat, Hunger, Fullness, Satiety are [0.99; 0.96], [0.98; 0.94], [0.97; 0.96], and [0.97; 0.97]

For the test set Dataset B with the DataName: Casein-low and Sucrose-high, satiety hormone measurements were available for GLP-1 and PYY but not for CCK. Therefore, CCK was predicted from GLP-1 and PYY using a separately trained (i.e. on dataset A) ANN. The ANN-predicted CCK responses averaged over 13 volunteers are shown in Fig. 3. Similarly, the VAS profiles for hunger, fullness, desire to eat, and satiety predicted from measured GLP-1, PYY, and predicted CCK are shown in Fig. 4. The correlation coefficient values were [Casein-low: 0.99; Sucrose-high: 0.96], [Casein-low: 0.98; Sucrose-high: 0.94], [Casein-low: 0.97; Sucrose-high: 0.96], and [Casein-low: 0.97; Sucrose-high: 0.97] for the Desire to eat, Hunger, Fullness, and satiety VAS responses, respectively. The predicted VAS response profiles showed higher satiety effects for Sucrose-high infusion compared to Casein-low infusion and that was expected.

In order to test which of the satiety hormones were most relevant in predicting the VAS profile, a test set, Casein-high, with all satiety hormones measured was chosen from Dataset B and its VAS responses were predicted. This was followed by iteratively removing one or two satiety hormones and creating a new test set. The VAS responses were then predicted for the newly created test set. Table 3 shows the results for the various combinations of hormone input data. As can be seen from the correlation coefficient averages, the test data with GLP-1 alone is as good as the rest.

**Table 3:** R-Correlation coefficient for predicted vs. measured VAS responses for different combinations of hormone input data as indicated (✓ = present, ✗ = absent) for casein-high infusion in Dataset B. VAS scores: D = Desire to eat; H = Hunger; F = Fullness; S = Satiety

Test data	GLP1	CCK	PYY	Correlation Coefficient (R)				
				D	H	F	S	Average
a.	✓	✗	✗	0.963	0.957	0.959	0.991	0.966
b.	✓	✓	✓	0.963	0.957	0.956	0.989	0.965
c.	✓	✓	✗	0.961	0.951	0.951	0.986	0.961
d.	✓	✗	✓	0.957	0.944	0.972	0.964	0.959
e.	✗	✓	✓	0.952	0.935	0.947	0.982	0.953
f.	✗	✓	✗	0.955	0.940	0.942	0.980	0.953
g.	✗	✗	✓	0.937	0.912	0.964	0.974	0.945

### Predicting VAS profiles for test sets with oral intake and gastric infusion

The training set Dataset C (Table 2) contained responses for all three satiety hormones and VAS response measurements until 180 min. For the test set Dataset D, GLP-1 was the only satiety hormone for which measurements were present, while the GLP-1 response and the VAS responses were measured during a much longer period i.e. until 270 min. Since the training set did not adequately cover the time range of the test set, the missing information was filled with the the autoregressive neural network as described in : Multilayer feed-forward Neural Network.

**Fig. 5:**
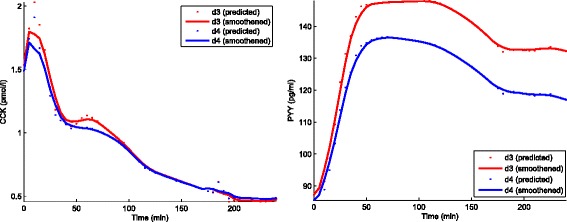
Predicted CCK and PYY responses. Predicted CCK (*left*) and PYY (*right*) responses averaged over 15 volunteers for Dataset D: d3, and d4

**Fig. 6:**
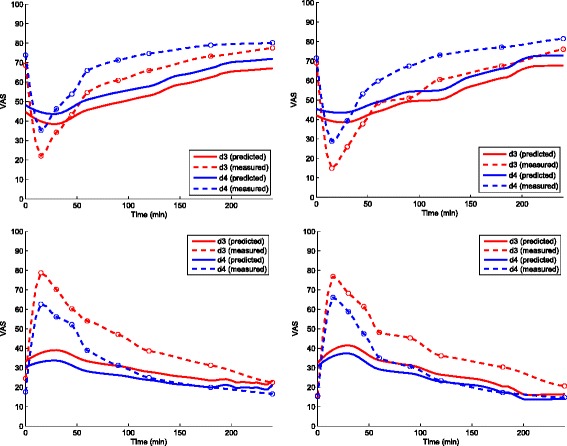
Measured and predicted VAS profiles. Measured and predicted VAS profiles (clockwise from top-left): Desire to eat, Hunger, Fullness and Satiety for Dataset D: d3, and d4 averaged over 15 volunteers. The prediction was based only on the GLP-1 data, from which CCK and PYY responses were predicted using separate ANNs. The respective correlation coefficients for d3, and d4 are [0.90; 0.87], [0.88; 0.85], [0.90; 0.91], and [0.93; 0.93]

The CCK and PYY responses predicted using an ANN trained on training set C, and using only GLP-1 responses as input are shown in Fig. 5. The predicted VAS score for desire to eat, hunger, fullness and satiety then predicted from measured GLP1 and predicted CCK, and PYY are shown in Fig. 6. The correlation coefficient values were [d3: 0.90; d4: 0.87], [d3: 0.88; d4: 0.85], [d3: 0.90; d4: 0.91], and [d3: 0.93; d4: 0.93] for the Desire to eat, Hunger, Fullness, and satiety VAS responses, respectively. The apexes (negative and positive peaks) of the predicted and the measured VAS responses did not match because the number of measurements available in the test set around the neighborhood of the apex were not sufficient enough to estimate the amplitude of the predicted VAS score in right proportion.

## Conclusion

Artificial neural networks (ANNs) provides an appropriate platform for modeling the complex input-output relationship between satiety hormones (GLP-1, PYY, and CCK) and satiety feelings in the brain as quantified by VAS scores (desire to eat, hunger, fullness and satiety). Application of ANN to predict satiety behavior produced satisfactory results. The obtained results upon systematically varying the combination of satiety hormone input data suggest that the presence of the satiety hormone GLP-1 is pivotal in determining the quality of the VAS response prediction. It also appeared that for achieving high quality VAS response prediction, the training sets should minimally contain measurements at time points when satiety hormones are expected to peak or valley in the test sets.

## Endnotes

^1^volunteers signed a written informed consent prior to participation, the study was conducted according to the principles of the Declaration of Helsinki, the Medical Ethics Committee of the University Hospital Maastricht and Maastricht University (METC azM/UM) approved the study.

^2^All subjects gave their written informed consent to participate in the study and the Central Denmark Region Committees on Health Research Ethics approved the study according to the Helsinki Declaration. The study was registered at Clinical trials. Gov ID: NCT01316354.
